# White matter abnormalities of corpus callosum in patients with bipolar disorder and suicidal ideation

**DOI:** 10.1186/s12991-019-0243-5

**Published:** 2019-09-10

**Authors:** Ran Zhang, Xiaowei Jiang, Miao Chang, Shengnan Wei, Yanqing Tang, Fei Wang

**Affiliations:** 1 Department of Psychiatry, First Affiliated Hospital, China Medical University, 155 Nanjing North Street, Shenyang, 110001 Liaoning People’s Republic of China; 2 Brain Function Research Section, First Affiliated Hospital, China Medical University, Shenyang, Liaoning People’s Republic of China; 3 Department of Radiology, First Affiliated Hospital, China Medical University, 155 Nanjing North Street, Shenyang, 110001 Liaoning People’s Republic of China; 4 Department of Geriatric Medicine, First Affiliated Hospital, China Medical University, Shenyang, Liaoning People’s Republic of China; 5 Department of Psychiatry, Yale University School of Medicine, New Haven, CT 06511 USA

**Keywords:** Bipolar disorder, Suicidal ideation, Corpus callosum, Diffusion tensor imaging

## Abstract

**Objective:**

Although many studies have shown that the corpus callosum (CC) may play an important role in bipolar disorder (BD) and suicide, the pathophysiological mechanism of BD underlying suicidal behavior is still unclear. This study aimed to explore the relationship between the CC, and BD and suicidal ideation using diffusion tensor imaging (DTI).

**Method:**

A total of 203 participants (47 BD patients with suicidal ideation, 59 with BD without suicidal ideation, and 97 healthy controls [HC]) underwent DTI scanning at a single site. We examined the white matter integrity of the CC in the three groups.

**Results:**

A comparison among groups showed that BD patients with suicidal ideation had significant lower fractional anisotropy (FA) values than those of BD without suicidal ideation and HCs in the body and genu of the CC, and FA values of BD without suicidal ideation were significantly lower than those of HCs. However, in the splenium of corpus callosum, no difference was found between BD without suicidal ideation and HCs.

**Conclusions:**

Our findings add to the evidence suggesting that the CC plays a key role in BD with suicidal ideation, especially with respect to the role of the genu and body of the CC subserving emotion regulation.

## Introduction

Suicide is a global public health problem that includes suicidal ideation, suicide attempts, and death by suicide. Suicidal ideation refers to thoughts, cognitions, and overt intent to kill oneself, which is of great significance in predicting suicidal behavior, as an early stage of suicide. Although people with suicidal ideation may not necessarily attempt suicide, suicidal ideation is still regarded as a risk factor [1]. Research has recognized that suicide is closely related to bipolar disorder (BD) [2]. An epidemiological study demonstrated that half of people who died of suicide had depression, and those with more serious mood disorders, such as BD, have a 20-fold increase in risk [3]. The risk of suicide among patients diagnosed with BD is as high as 6% over 20 years, while self-harm occurs in 30–40% [4].

Many magnetic resonance imaging (MRI) studies have been performed trying to reveal the underlying neuropathological mechanism of suicide in BD. Earlier studies focused on white matter (WM) abnormalities of BD patients with suicidal behavior [5–8]. In addition, the gray matter volume of BD who have suicidal behavior was shown to be decreased in the prefrontal cortex [9, 10], lingual gyrus, and right cuneus [11], and significantly increased in the right rostral anterior cingulate cortex [12]. A multimodal neuroimaging study [13] suggested that there are gray matter volume decreases, and structural and functional connectivity abnormalities in the ventral frontolimbic neural system in adolescents and young adults with BD who attempt suicide. Despite the many studies of suicide in patients with BD, a specific suicide-related biological basis of BD remains unclear, and few studies have focused on suicidal ideation, which is an early stage of suicidal behavior.

The corpus callosum (CC) is the largest interhemispheric commissure for higher cognitive functions associated with emotion, which involves sustaining attention [14], approach-related motivation and behavior [15], inhibition and excitation of the contralateral hemisphere [16], affective prosody [17], and memory [18]. Previous studies have linked alterations in the CC with the risk of suicide and the pathophysiology of BD. A review of diffusion tensor imaging (DTI) studies in BD suggested that regions underlying inter-hemispheric communication such as the CC may play a major role in the pathophysiology of bipolar disorder [19]. Moreover, a recent DTI study [20] also found that the genu and body of the CC are altered in non-suicide attempters with BD and the splenium is specifically altered in those who attempt suicide. Some studies of BD and suicide attempts failed to confirm the same findings [21, 22]; however, the authors explained that this might have been caused by the sample size, selection of the samples, medication, and state of the disease, among other factors. The close relationship between suicide risk in patients with BD and variation in the CC is, however, recognized by most researchers.

Therefore, we consequently inferred that the CC is the key to understand the specific region alteration in BD. To investigate the role of the CC in the mechanism of suicide in BD, we chose the CC as the region of interest to detect the WM integrity of the CC in BD patients with suicidal ideation compared with that in BD without suicidal ideation and healthy people. We hypothesized that the WM tracts of the BD patients with suicidal ideation in the CC would be decreased than those without suicidal ideation, especially in the anterior CC areas.

## Method

### Subjects

One hundred and six patients with BD and 97 healthy controls (HCs), aged 15–50 years, were involved in this study. Written informed consent was obtained from all participants in the study, and this study was approved by the Ethics Committee of the first affiliated Hospital of China Medical University. All patients were recruited from the inpatient department of the Shenyang Mental Health Center and the outpatient clinic of the Department of Psychiatry of the First Affiliated Hospital of China Medical University, Shenyang, China. The subjects were considered to have “BD” if they met the diagnostic criteria of the Structured Clinical Interview for the Diagnostic and Statistical Manual of Mental Disorders (DSM)-IV Axis I disorders for those ≥ 18 years old and the Kiddie Schedule for Affective Disorders and Schizophrenia for School-Age Children-Present and Lifetime Version in those < 18 years old. The exclusion criteria were (1) no history of other Axis I mental disorders; (2) no history of major somatic disease, especially diseases that may be related to changes in brain tissue, such as hypertension or diabetes; (3) no history of abnormalities of the nervous system, including major head trauma, epilepsy, cerebrovascular disease, brain tumors, or neurodegenerative diseases; and (4) no contraindications for MRI.

HCs were recruited through advertisements and matched the BD group for age, sex, and number of years in education. No healthy participants had a history of suicide attempts or suicidal ideation, current Axis I disorder, or history of Axis I disorders in their first-degree relatives according to a detailed family history; the other exclusion criteria were the same as the participants with BD.

### Clinical assessment

Suicidal ideation severity was assessed using the 19-item Beck Scale for Suicide Ideation [23]. Based on their scores on the Beck Scale for Suicide Ideation, we divided all patients with BD into a BD with suicidal ideation group and BD without suicidal ideation group and excluded any healthy participants who had suicidal ideation.

The severity of clinical symptoms in all participants was evaluated using the 17-item Hamilton Depression Rating Scale (HAMD_17), Hamilton Anxiety Scale (HAMA), and Young Manic Rating Scale (YMRS). Detailed demographic and clinical characteristics of participants are outlined in Table 1.

**Table 1:** Demographic and clinical characteristics of participants

Characteristic	BD with suicidal ideation (*N* = 47)		BD without suicidal ideation (*N* = 59)		HC (*N* = 97)		Statistics	
	Mean	SD	Mean	SD	Mean	SD	*F/t*	*p*
Age	26.53	8.02	25.15	7.69	24.32	8.35	1.189	0.307
Education, years	13.40	2.92	12.19	2.83	12.92	2.49	2.796	0.063
Duration, month	43.54	49.14	42.03	53.39	NA	NA	0.134	0.894
HAMD-17	*N* = 45		*N* = 58				0.449	0.655
	11.51	10.34	10.66	9.00	NA	NA		
HAMA	*N* = 45		*N* = 53				1.363	0.176
	11.69	10.91	9.04	8.33	NA	NA		
YMRS	*N* = 46		*N* = 56				0.203	0.840
	6.04	8.96	5.70	8.29	NA	NA		

Characteristic	BD with suicidal ideation (*N* = 47)		BD without suicidal ideation (*N* = 59)		HC (*N* = 97)		Statistics	
	*N*	%	*N*	%	*N*	%	*χ* ^2^	*p*
Sex, male	16	34.04	26	44.07	47	48.45	2.672	0.263
Medications, yes	37	78.72	36	61.02	NA	NA	3.825	0.050
First episode, yes	19	40.43	31	52.54	NA	NA	1.765	0.184
Mood state at scan					NA	NA	3.006	0.222
Euthymic	10	21.28	7	11.87	NA	NA		
Depressed	19	40.43	33	55.93	NA	NA		
Stable	18	38.29	19	32.20	NA	NA		

Data are presented as *n* (%) or mean (standard deviation)

*BD* bipolar disorder, *HC* healthy control, *N/A* not applicable, *HAMD_17* 17-item Hamilton Depression Rating Scale, *HAMA* Hamilton Anxiety Scale, *YMRS* Young Manic Rating Scale

### Image acquisition

All participants consented to a DTI scanning session using a GE Sigma HDX 3.0-T superconductive magnetic resonance scanner with a standard 8-channel head coil at the First Affiliated Hospital of China Medical University, Shenyang, China. The scanning parameters were as follows: TR/TE = 17,000/85.4 ms, image matrix = 120 × 120, FOV = 240 × 240 mm^2^, 65 contiguous slices of 2 mm without gap, 25 noncollinear directions (*b* = 1000 s/mm^2^), together with axial acquisition without diffusion weighting (*b* = 0), and voxel size = 2.0 mm^3^.

### Image processing

Brain images were processed using the Pipeline for Analyzing braiN Diffusion imAges (PANDA) (http://www.nitrc.org/projects/panda), which is a MATLAB toolbox for the pipeline processing of diffusion MRI [24]. Processing proceeded as follows: first, the voxel-wise diffusion tensor matrix was calculated for each subject in the native space. Next, diagonalization was performed to yield three pairs of eigenvalues and eigenvectors. Based on the three eigenvalues, FA values were computed on a voxel-by-voxel basis. Specifically, the FA image of each subject was nonlinearly registered to the FMRIB58_FA template in a standard Montreal Neurological Institute (MNI) space via spatial normalization (voxel size: 1 mm × 1 mm × 1 mm). The mean of all aligned FA images was then calculated. Finally, FA images were smoothed with a 6-mm full width at half maximum Gaussian filter.

### Region of interest

To compare the structural changes in BD patients with and without suicidal ideation, we selected the CC as the region of interest (ROI). The CC mask was defined according to JHU ICBM-DTI-81 WM labels atlas [25] contained in MRIcron, which included the genu, body, and splenium of the CC.

### Statistical analysis

We compared the demographic data of BD patients with and without suicidal ideation and HCs using Student’s *t*-tests, one-way analyses of variance (ANOVA), and Chi-square tests. All statistical analyses were conducted using IBM SPSS statistical software for Windows, version 22.0 (Armonk, NY, USA). Categorical variables are described using frequencies and proportions. Continuous variables are presented as the mean ± standard deviation.

Further, to investigate the main effect of group, we performed an ANOVA among BD with suicidal ideation group, BD without suicidal ideation groups and HC group using the CC mask. Statistical significance was determined at level of *p *< 0.001 after Gaussian Random Field (GRF) correction, corresponding to a threshold of 20. All statistical analyses were performed using the Statistical Parametric Mapping (SPM) software (SPM8; The Wellcome Department of Cognitive Neurology) and Data Processing and Analysis for Brain Imaging software (DPABI, DPABI_V1.2_141101, http://rfmri.org/dpabi), and age, sex, and years of education as covariates.

In post hoc analyses, FA values were extracted for each cluster with significant differences among the three groups, and groups then be compared pair-wise. Significance level was set at *p *< 0.05 (least significant difference test; LSD).

## Results

### Clinical results

Table 1 describes the demographic and clinical data of the three groups. No significant differences in age (*F *= 1.189, *p *= 0.30; < 18 years), sex (*χ*^2^ = 2.672, *p *= 0.263), or years of education (*F *= 2.796, *p *= 0.063) were found among groups. Among our samples, 35 participants younger than 18 years have been included into our study (BD with suicidal ideation group: *n* = 4, 0.09%; BD without suicidal ideation group: *n* = 10, 0.17%; HC group: *n* = 21, 0.22%). In our samples, there were no significant differences between the BD with suicidal ideation group and BD without suicidal ideation group in the duration of disease, medications, type of first episode, mood state at scan, and HAMD, HAMA, and YMRS scores.

### Group differences within DTI images

**Fig. 1:**
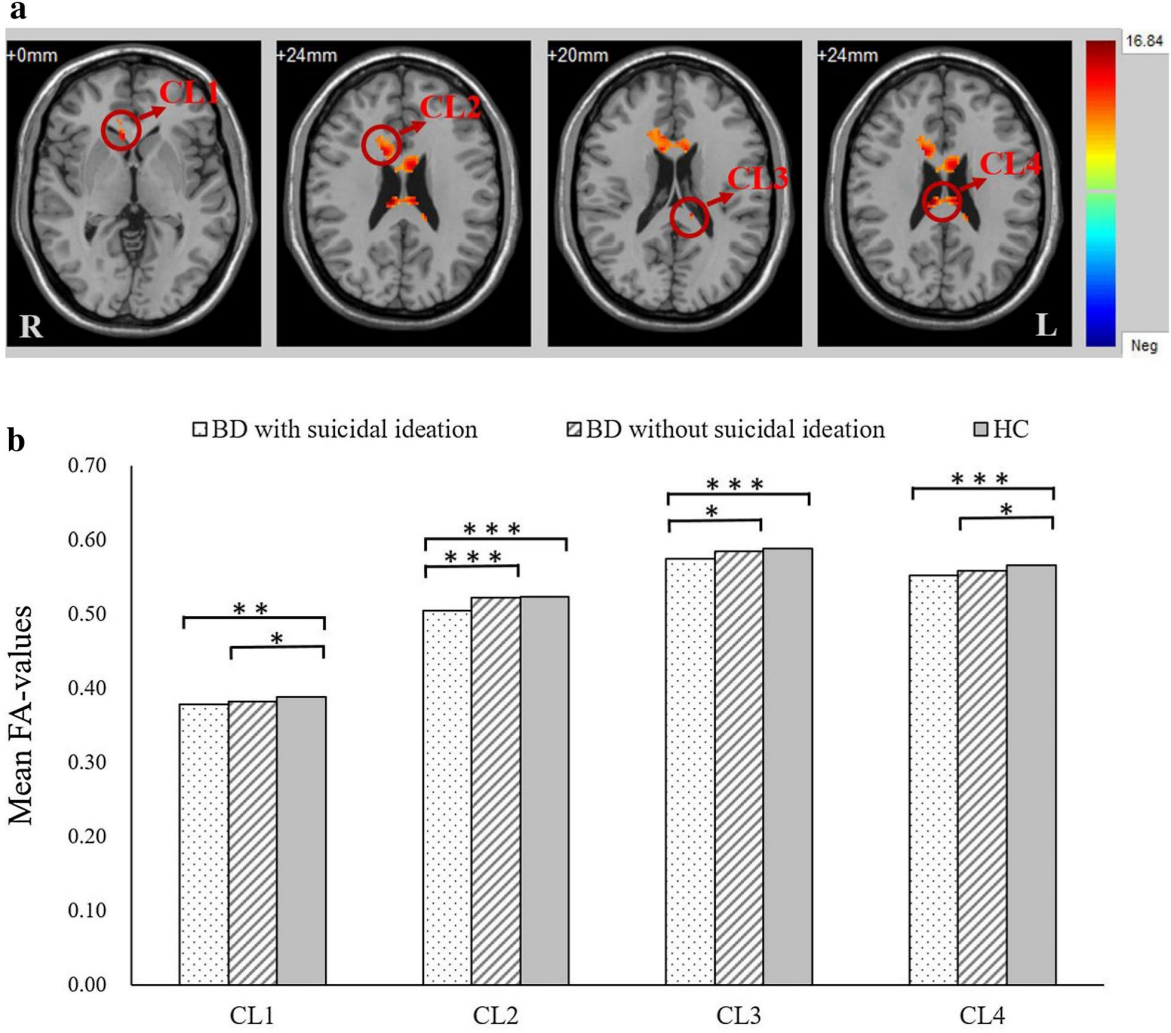
CL 1: Genu of corpus callosum, CL 2: Body of corpus callosum/genu of corpus callosum, CL 3: Splenium of corpus callosum, CL 4: Body of corpus callosum. **a** Significant differences in fractional anisotropy values among BD patients with suicidal ideation, BD patients without suicidal ideation, and healthy controls. Significant at *p *< 0.001 after GRF correction. **b** Post hoc analysis of fractional anisotropy values with significant differences among the three groups. ***p < 0.001 level, **p < 0.01 level, LSD test

In our samples, the inter-group comparisons demonstrated significant differences in four clusters within the body, splenium, and genu of the CC (*p *< 0.001, GRF corrected). In particular, the patients with BD had lower FA values than HCs in all four clusters, a difference which was common to both BD with and without suicidal ideation groups. Post hoc analysis revealed that the BD with and without suicidal ideation groups had lower FA values in the body and genu of the CC (CL 1 and CL 4) than HCs (*p *< 0.05, LSD test), but there was no significant difference between the BD with and without suicidal ideation groups (*p *> 0.05, LSD test). The FA values of the BD with suicidal ideation group were much lower than those of the BD without suicide ideation group and HCs in the body, splenium, and genu of the CC (CL 2 and CL 3), but there was no significant difference (*p *> 0.05, LSD test) between the BD without suicidal ideation group and HCs (Fig. 1 and Table 2).

**Table 2:** Comparison of FA values in the corpus callosum among BD patients with suicidal ideation, BD patients without suicidal ideation and healthy controls

Index	WM labels^a^	Cluster size	Montreal neurological institute coordinates			F*
			*x*	*y*	*z*	
Cluster 1	Genu of corpus callosum	21	8	22	0	12.72
Cluster 2	Body of corpus callosum	642	14	12	24	13.56
	Genu of corpus callosum					
Cluster 3	Splenium of corpus callosum	22	− 12	− 34	20	11.07
Cluster 4	Body of corpus callosum	100	10	− 26	24	16.84

* Significant at *p *< 0.001 corrected by GRF correction

^a^WM labels are provided in accordance with the ICBM-DTI-81 White Matter Labels Atlas

## Discussion

Our results show that BD patients with suicidal ideation have significantly lower FA values in the CC than BD patients without suicidal ideation and HCs. Indeed, the FA values of BD patients with suicidal ideation were lower than those of HCs. In accordance with the law of diminishing returns, comparing the FA values found that BD patients with suicidal ideation have the most severe white matter damage, followed by BD patients without suicidal ideation, which suggests that the presence of suicidal ideation is related to this change.

The CC connects the left and right cerebral hemispheres and enables interhemispheric communication. Also, the genu and anterior CC are thought to be relevant in the regulation of emotion [26]. Our finding that the reduction in FA in the CC associated with BD was in the body and genu of the CC is consistent with previous studies. For instance, a meta-analysis [27] revealed that BD show reduced callosal areas in volume in comparison with healthy volunteers. Furthermore, a reduction in the anterior CC (including the genu, posterior body, and isthmus) area, was previously reported [28, 29], in addition to a reduction in the central CC area [30]. Accordingly, we thought that reduced callosal areas may lead to altered interhemispheric communication, participant in the pathophysiological mechanism of BD.

Moreover, we found a significant reduction of FA values in the whole CC in the BD with suicidal ideation group compared to those in the BD without suicidal ideation group and HCs. No significant differences were observed between the BD without suicidal ideation group and HCs, which suggests an association between the CC and suicidal ideation. It has been proposed that abnormalities in hemispheric connectivity are associated with suicidal behavior [30] and that the CC is believed to play a more important role in interhemispheric connections. Studies of suicide have directly highlighted that FA value reductions in the CC are associated with suicidal behaviors in agreement with our findings. A additional study have also linked decreased CC areas to elderly and other populations who have suicidal behavior [31, 32]. These studies support our findings that the observed reduced FA values in the CC may reveal the structural brain alterations underlying suicidal ideation.

In addition, the results indicate that the reduction in FA values in the body and genu of the CC was closely related to both the pathology of BD and the existence of suicidal ideation, while decreased FA values in the splenium of the CC may be a trait of BD. A previous study that also used the CC as the ROI [21] found that suicidal BD patients with higher impulsivity had a smaller anterior genu area, which supports our finding that an atrophied anterior CC region in patients with BD may lead to suicidal behaviors, even if they only currently exhibit suicidal ideation. However, there was a study [33] that did not explore the differences in the CC between suicide attempters and non-attempters that raised the idea that central parts of the CC may be a biomarker of bipolarity, which may be because the sample contained two diagnoses, BD and MDD, and is inconsistent with the single population of this study. Interestingly, a similar study in female suicide attempters in France was performed [20] and reached a conclusion diametrically opposed to ours. Therefore, we may need to further study the brain structure alterations of female Chinese BD patients with suicidal behavior or include more samples to verify the results. Generally, we identified that there are certainly some connections between the atrophied CC region and BD with suicidal behavior, especially related to the anterior CC. This led us to make the deduction that the reason for suicidal ideation in BD may be that CC atrophy leads to impaired higher cognitive function associated with emotions, such as the regulation of emotion.

Most of these studies focused on damage to the cerebral circuitry in patients with BD who attempted suicide; however, little attention was paid to BD with suicidal ideation. We preliminarily focused on suicidal ideation with the idea that it is the first step to suicide [34] and found that BD patients with suicidal ideation have lower FA values in the body and genu of the CC. This suggests that impairments in the CC exist in the early stages of suicide risk.

Certainly, the limitations of this study should also be considered. Firstly, there were a small number of subjects included in this study. Thus, the characteristics of the CC in BD patients with suicidal behavior still need to be validated in a larger sample. Secondly, our sample size was not sufficient to analyze differences between patients with type I and type II BD; later studies should examine the relationship with BD type to produce more convincing results. Another limitation was medication is hardly to deal with. Although many studies showed no significant effects of medications on FA, a few studies suggested that those patients with BD treated with a mood stabilizer (versus not taking mood stabilizers) [35] or lithium (versus not taking lithium) [36, 37] may contribute to increased FA values in WM tracts. In the present study, we were not sure how medication affect those DTI measurements and how differ in WM tracts among groups with different drug abuse merits. In the future, we need to recruit more nonmedicated patients with BD to explore this problem (Additional file 1: Table S1).

In summary, this study proved that brain alterations in the body and genu of the CC are associated with suicidal ideation in BD, while changes in the splenium of the CC may be a pathological trait of BD. Moreover, our study provided preliminary imaging evidence for the early identification and prevention of suicide risk in BD and presented a good example for further study of functional and structural features in BD with suicidal ideation and attempts. In the future, large samples and longitudinal studies are needed to identify the specific imaging markers associated with suicidal risk in BD. Additional studies of the role of the corpus callosum in suicidal behavior in BD are also warranted.

## Supplementary information

**Additional file 1: Table S1.** The medication status of the two BD groups.

## Data Availability

The data sets used and/or analyzed during the current study are available from the corresponding author on reasonable request.

## Abbreviations

CC: corpus callosum
BD: bipolar disorder
DTI: diffusion tensor imaging
HC: health controls
FA: fractional anisotropy
MRI: magnetic resonance imaging
WM: white matter
DSM-IV: Diagnostic and Statistical Manual of Mental Disorders
HAMD_17: the 17-item Hamilton Depression Rating Scale
HAMA: Hamilton Anxiety Scale
YMRS: Young Manic Rating Scale
PANDA: the Pipeline for Analyzing braiN Diffusion imAges
MNI: Montreal Neurological Institute
ROI: region of interest
ANOVA: analyses of variance
SPM: Statistical Parametric Mapping Software
DPABI: Data Processing and Analysis for Brain Imaging software
GRF: Gaussian Random Field
LSD: least significant difference test
