# Supplementary material for: White matter abnormalities of corpus callosum in patients with bipolar disorder and suicidal ideation
Source: Ann Gen Psychiatry. 2019 Sep 10;18:20. doi: 10.1186/s12991-019-0243-5 (PMC6737682; doi:10.1186/s12991-019-0243-5)
Supplement: Supplementary file 1 — Additional file 1: Table S1. The medication status of the two BD groups. [file 12991_2019_243_MOESM1_ESM.docx]

**Additional Material**

We have showed the medication status of patients with BD according to the Reviewer’s comments in Table S1.

**Table S1. The medication status of the two different BD groups**

| Characteristic | BD with suicidal ideation(N=47) | | BD without suicidal ideation(N=59) | |
| --- | --- | --- | --- | --- |
|  | n | % | n | % |
| Unmedicated | 10 | 21.28 | 23 | 38.98 |
| Medications |  |  |  |  |
| Antidepressants | 19 | 40.43 | 15 | 25.42 |
| Antipsychotics | 20 | 42.55 | 21 | 35.59 |
| Antianxiety drugs | 6 | 12.77 | 4 | 0.07 |
| Valproic Acid | 22 | 46.80 | 26 | 44.07 |
| Lithium carbonate | 1 | 0.02 | - | - |
| Other mood stabilizers | 1 | 0.02 | - | - |
